# Identifying and addressing mentorship gaps in European trauma and emergency surgical training. Results from the Young European Society of Trauma and Emergency Surgery (yESTES) mentorship survey

**DOI:** 10.1007/s00068-024-02610-y

**Published:** 2024-08-09

**Authors:** Stefano Piero Bernardo Cioffi, Laura Benuzzi, Marit Herbolzheimer, Enrico Marrano, Gabriele Bellio, Wouter Pieter Kluijfhout, Frans-Jasper Wijdicks, Annika Hättich, Peep Talving, Eileen Bulger, Jonathan Tilsed, Diego Mariani, Cristina Rey Valcarcel, Shahin Mohseni, Susan Brundage, Carlos Yanez, Jan P. A. M. Verbruggen, Frank Hildebrand, Inger B. Schipper, Christine Gaarder, Stefania Cimbanassi, Hayato Kurihara, Gary Alan Bass

**Affiliations:** 1 Department of Surgery, University of Rome Sapienza, Rome, Italy; 2 General Surgery Trauma Team, Niguarda Hospital, Milan, Italy; 3 Young-ESTES, European Society for Trauma and Emergency Surgery, St. Polten, Austria; 4 Department for Trauma Surgery, BG Trauma Center Murnau, Murnau, Germany; 5 Department of General Surgery, Germans Trias I Pujol University Hospital, Barcelona, Badalona Spain; 6 Emergency Surgery Unit, IRCCS Fondazione Ca’ Granda Ospedale Maggiore Policlinico, Milan, Italy; 7 Department of Traumasurgery, OLVG Amsterdam, Amsterdam, The Netherlands; 8 Department of Trauma Surgery, Diakonessenhuis Hospital Utrecht, Utrecht, The Netherlands; 9 Department for Trauma and Orthopaedic Surgery, University Hospital Hamburg Eppendorf, Hamburg, Germany; 10 Institute of Clinical Medicine, University of Tartu, University Hospital, Puusepa 8, Tartu, Estonia; 11 Harborview Medical Center, University of Washington, Seattle, WA USA; 12 Hull Royal Infirmary, Anlaby Road, Hu3 2Jz, Hull, England UK; 13 UEMS Division of Emergency Surgery, Liverpool, UK; 14 Asst Ovest Milanese, Chirurgia Generale E Urgenza, Legnano, Italy; 15 Hospital General Universitario Gregorio Marañón, Madrid (HGGM), Spain; 16 Department of Surgery, Sheikh Shakhbout Medical City (SSMC), Abu Dhabi, United Arab Emirates; 17 Department of Surgery, R Adams Cowley Shock Trauma Center, Baltimore, USA; 18 General and Acute Care Surgery Department, San Jorge University Hospital, Huesca, Spain; 19 Department of Trauma Surgery, Maastricht University Medical Center, Maastricht, The Netherlands; 20 Department of Orthopaedics Trauma and Reconstructive Surgery, University Hospital RWTH, Aachen, Germany; 21 Trauma Surgery Department, University of Leiden, Leiden University Medical Center, Leiden, The Netherlands; 22 Department of Traumatology, Oslo University Hospital Ullevål (OUH U), Olso, Norway; 23 Department of Surgical Pathophysiology and Transplantation, State University of Milan, Milan, Italy; 24 State University of Milan, Milan, Italy; 25 Division of Traumatology, Surgical Critical Care and Emergency Surgery, Perelman School of Medicine of the University of Pennsylvania, Philadelphia, USA; 26 Center for Peri-Operative Research and Transformation (C-PORT), University of Pennsylvania, Philadelphia, USA

**Keywords:** Mentorship, Trauma surgery, Emergency general surgery, Non-technical skills, Surgical education, Gender gap, Early-career surgeons

## Abstract

**Purpose:**

European training pathways for surgeons dedicated to treating severely injured and critically ill surgical patients lack a standardized approach and are significantly influenced by diverse organizational and cultural backgrounds. This variation extends into the realm of mentorship, a vital component for the holistic development of surgeons beyond mere technical proficiency. Currently, a comprehensive understanding of the mentorship landscape within the European trauma care (visceral or skeletal) and emergency general surgery (EGS) communities is lacking. This study aims to identify within the current mentorship environment prevalent practices, discern existing gaps, and propose structured interventions to enhance mentorship quality and accessibility led by the European Society for Trauma and Emergency Surgery (ESTES).

**Methods:**

Utilizing a structured survey conceived and promoted by the Young section of the European Society of Trauma and Emergency Surgery (yESTES), we collected and analyzed responses from 123 ESTES members (both surgeons in practice and in training) across 20 European countries. The survey focused on mentorship experiences, challenges faced by early-career and female surgeons, the integration of non-technical skills (NTS) in mentorship, and the perceived role of surgical societies in facilitating mentorship.

**Results:**

Findings highlighted a substantial mentorship experience gap, with 74% of respondents engaging in mostly informal mentorship, predominantly centered on surgical training. Notably, mentorship among early-career surgeons and trainees was less reported, uncovering a significant early-career gap. Female surgeons, representing a minority within respondents, reported a disproportionately poorer access to mentorship. Moreover, while respondents recognized the importance of NTS, these were inadequately addressed in current mentorship practices. The current mentorship input of surgical societies, like ESTES, is viewed as insufficient, with a call for structured programs and initiatives such as traveling fellowships and remote mentoring.

**Conclusions:**

Our survey underscores critical gaps in the current mentorship landscape for trauma and EGS in Europe, particularly for early-career and female surgeons. A clear need exists for more formalized, inclusive mentorship programs that adequately cover both technical and non-technical skills. ESTES could play a pivotal role in addressing these gaps through structured interventions, fostering a more supportive, inclusive, and well-rounded surgical community.

**Supplementary Information:**

The online version contains supplementary material available at 10.1007/s00068-024-02610-y.

## Introduction

The journey toward expertise in surgery encompasses more than the attainment of technical prowess and theoretical knowledge. In Europe, the trajectory for clinicians specializing in the multifaceted and often overlapping realms of visceral and skeletal major trauma care (treatment of the severely-injured patient) and emergency general surgery (treatment of acute surgical conditions of the abdomen, soft tissues, and non-traumatic surgical emergencies) remains non-uniform, marred by a lack of consensus and divergent educational frameworks attributable to profound organizational and cultural disparities across countries [1]. These disparities, and the lack of a standard training regimen, underscore the necessity for a tailored mentorship structure capable of bridging the resultant gaps in surgical education and practice.

Mentorship, a cornerstone of medical education, transcends conventional technical training by fostering an environment conducive to guiding research and non-technical skill (NTS) acquisition [2]. Mentees, particularly those transitioning to independent practice, have a complex array of needs that often cannot be met by a single mentor. Dynamic training needs necessitates the adoption of “mosaic mentorship” which draws on the complementary experience, insights, and support from multiple mentors, acknowledging the changing nature of their needs over time [3]. These encompass a suite of competencies such as surgical heuristics [4], patient communication, and intra-operative decision-making, pivotal to success in the nuanced landscape of trauma and emergency surgical care. The mentor–mentee relationship, therefore, serves as the crucible within which aspiring surgeons can be forged, informed by the wisdom and insight of seasoned practitioners, thereby enhancing their surgical independence and confidence [5, 6]].

However, the existing mentorship paradigms within Europe’s trauma and emergency surgery domains manifest as fragmented and predominantly informal constructs, predominantly hinging on serendipitous interactions rather than structured, intentional guidance. This approach, while beneficial, often falls short of addressing the comprehensive needs of young surgeons, particularly when navigating the complex interplay of surgical performance pressures emanating from the operative environment, patient crises, and intrinsic surgeon-related stressors. This fragmented mentorship landscape conspicuously lacks a cohesive framework addressing the integral components of surgical mentorship, particularly in areas pertaining to critically ill injured and non-injured surgical patients. The resultant void not only hampers the holistic development of emerging surgeons but also attenuates the potential for optimized patient care outcomes [1].

The Young European Society of Trauma and Emergency Surgery (yESTES) Committee represents surgeons in the terminal portion of their training or in transition to independent practice [7]. Recognizing these challenges, we conducted a comprehensive survey of ESTES members, aimed at delineating the current state of mentorship within this specialized field. The initiative sought to unravel the prevailing trends, experiences, and deficiencies characterizing mentorship across European nations, with an eye towards crafting actionable strategies for the European Society for Trauma and Emergency Surgery (ESTES). The ultimate objective lies in fortifying formal mentorship avenues, fostering a robust international academic and surgical network, and catalyzing a supportive ecosystem for those embarking on or navigating the intricacies of a surgical career in trauma and emergency settings.

We present a narrative on the mentorship *status quo*, based on the insights garnered from the yESTES mentorship survey. We aim to unravel the layers of mentorship experiences as perceived and reported by contemporary surgeons, identify salient gaps afflicting early-career surgeons and underrepresented trainee groups such as women, and highlight the undervalued yet crucial role of non-technical skills in surgical mentorship. Furthermore, it aims to spotlight the role that surgical societies like ESTES can play in ameliorating these gaps, thereby enhancing the mentorship landscape for trauma and emergency surgeons across Europe.

The driving hypothesis of the study is that mentorship in trauma and emergency surgery among ESTES members is affected by the heterogeneity inherent to different training systems in Europe. Thus, mentor–mentee relationships may not be guided by formal or institutional programs and not adequately oriented on diversity and equity, and not equally addressing the special needs of surgeons at different career stages.

## Methods

This electronic survey study was performed during the period of March 1st through April 30th 2023 among the registered individual members of ESTES. One mid-term e-mail reminder was sent to the members. The population of interest was constituted by trainees or surgeons dedicated to at least one of the following fields: EGS, visceral trauma and skeletal trauma in the context of polytrauma. Participation was anonymous, confidential, voluntary and free from financial incentive. Thus, we did not request Institutional Review Board (IRB) study approval.

### Outcomes

The primary outcome was the presence of mentorship relationships among survey respondents. Secondary outcomes included the nature of mentorship (formal vs. informal), areas of mentorship focus (technical skills, non-technical skills), the impact of mentorship on career development and the identification of the role of surgical societies and their potential impact.

### Survey design

A 29-question electronic survey instrument was developed by yESTES Committee [7] in consultation with senior members of the Society with a track record of surgical mentorship. Because no suitable validated survey instruments exist, we created a survey instrument with the intention of qualitatively exploring mentorship experiences and attitudes in our study populations. MEDLINE, Scopus, Embase, and Google Scholar were queried using the terms mentorship”, “surgery”, “survey”. The survey was divided into four parts: respondent demographics, mentorship experience, perceptions on the mentor–mentee relationship and opinions on the role of surgical societies, research gaps and future interventions. The complete survey is reported in the Supplementary materials, S1. The survey was conducted following the CHERRIES (Checklist for Reporting Results of Internet E-Surveys) method [8]. A practical guide published in JAMA Surgery on survey research was also followed [9]. The survey was administered through SurveyMonkey (SurveyMonkey Inc., San Mateo, California, USA) via email to 413 junior and senior members of ESTES from the member mailing list of the society.

### Data management

To prevent multiple data entries from the same respondent the IP address was used to automatically identify potential duplicates. Data was anonymized and stored securely.

### Statistical analysis

#### Hypothesis testing

We tested the null hypothesis that there is no difference in mentorship experiences across gender (women, men), specialty (visceral trauma, skeletal trauma, EGS), career stage (resident, surgeons in transition to independent practice, surgeons in established practice) and geographic location among ESTES members. Descriptive statistics summarized respondent demographics and survey responses. Differences were assessed using chi-squared tests for categorical variables and ANOVA or Kruskal–Wallis tests for continuous variables, depending on the normality of the data. Statistical analysis was performed with jamovi v. 2.5.2 (2024, https://www.jamovi.org.) Statistical significance for frequentist tests was set at an alpha level of 0.05. Sample size calculation was not performed.

### Authorship strategy

Study authors were jointly selected by the first and last author based on several defining characteristics, including: prominence in the fields of Traumatology and Emergency Surgery, service to ESTES (including organization leadership Council service, as well as prior or current service within the organization, with special reference to the Young ESTES project), expertise in delivering or assessing education or educational methods, and relevant peer-reviewed manuscript publication history. The selection process actively strove to appropriately represent national, career stage and gender diversity within the expert grouping.

## Results

### Demographics

**Fig. 1:**
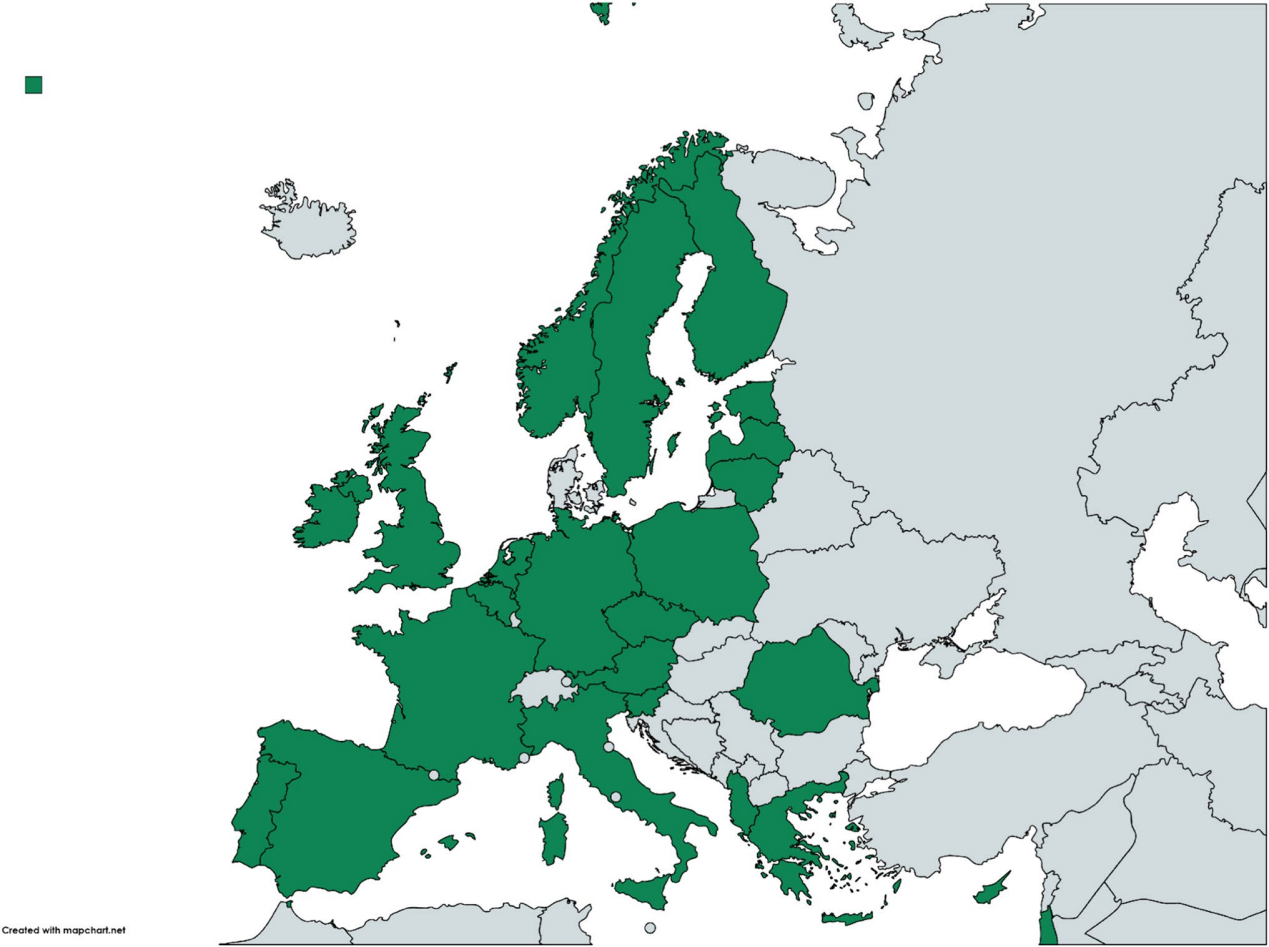
Geographical distribution of the respondents covering most of the western, central and northern European area, with a low rate of involvement for eastern European countries

One-hundred and twenty-three physicians completed the survey. The response rate was 29.8%% (123/413). 28.5% of survey respondents were female. The median age was 39 years, Interquartile Range (IQR) 31.5–47.5. Among respondents, 31.5% were surgical trainees, 24.4% were surgeons in transition to independent practice and 45.5% surgeons in established practice. Considering the main clinical focus, 45.5% were dedicated to visceral trauma, 43.9% to emergency general surgery and 10.6% to skeletal trauma. The geographical distribution of the respondents is shown in Fig. 1.

### Mentorship experience

Seventy-four point eight percent acknowledged having received mentorship to date, of whom 68.7% benefited from mosaic mentorship. Sixty percent met their first mentor during the residency program and the relationship was informal for all the respondents except for 2% who took part in a formal mentorship program. None of the participants had the chance to meet their mentor through a surgical society meeting.

### Perceptions on the mentor–mentee relationship

**Fig. 2:**
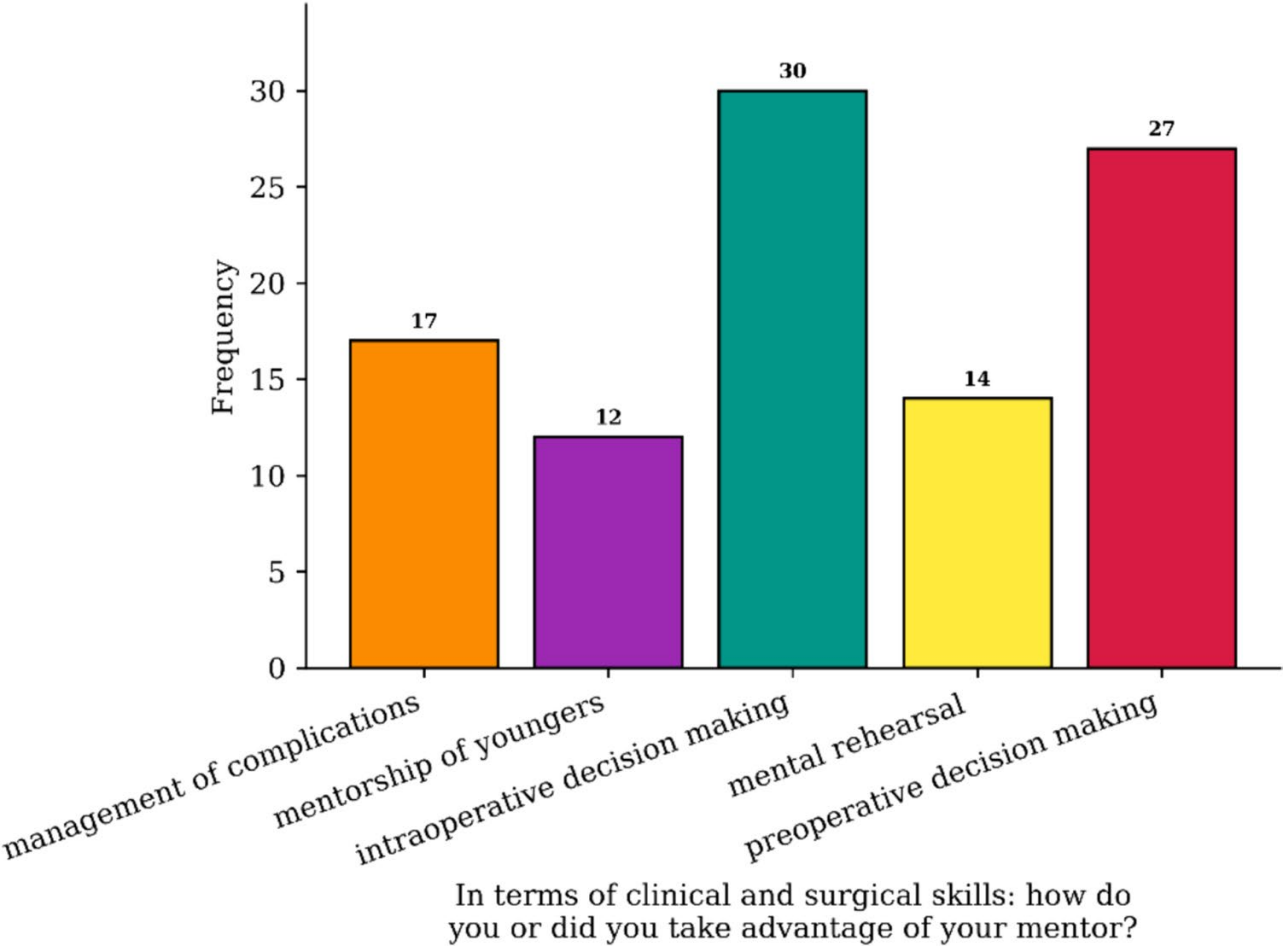
The bar chart shows how most of the mentees interviewed benefited from intra and peri-operative support during the surgical decision-making process. Mentorship of young people: learning how to be a mentor. Mental rehearsal: mental recovery from difficult clinical situations and surgical complications

Sixty percent of the respondents identified strong interpersonal skills as important qualities for a mentee, enabling them to derive the most benefit from engagement with a potential mentor. Seventy-two percent identified surgical training as the ideal career time-frame to first identify a mentor. We explored the main scenarios in which clinical and surgical mentorship impacts, and peri- and intra-operative surgical decision making were the most addressed. The results of this question are reported in Fig. 2.

### The role of surgical societies, research gaps and future interventions

Ninety-four percent of the participants felt that mentoring programs should be formally supported by institutions as universities or scientific societies. Table 1 summarized the results of the prioritization process made by the ESTES member participants on initiatives to foster mentorship in Europe. Traveling fellowships, remote and in-person research fellowships were identified as implementation priorities. The survey responses are reported in the supplementary materials 2, S2.

**Table 1:** Member-identified priorities for dedicated initiatives to foster mentorship in trauma and emergency surgery in Europe

Initiative	Priority class						
	1st	2nd	3rd	4th	5th	6th	7th
Remote clinical mentorship programs	**23.3%**	17.7%	14.0%	7.4%	15.8%	16.8%	4.6%
Remote research mentoring programs	15.8%	**22.4%**	16.8%	13.0%	17.7%	10.2%	3.7%
Traveling clinical fellowships	**26.1%**	18.6%	25.2%	14.0%	8.4% 9	5.6% 6	1.8%
Research fellowships	6.5%	11.2%	**26.1%**	**28.9%**	16.8%	10.2%	0%
Surgical hands-on courses	**23.3%**	15.8%	10.28%	11.2%	**22.4%**	12.1%	4.6%
Summer school in medical writing	0%	1.8%	0.9%	9.35%	6.54%	**40.1%**	41.1%
Non- technical skills courses	4.6%	12.1%	6.5%	15.8%	12.1%	4.6%	**43.9%**

Most rated initiatives per class are bold and underlined. The answers to the survey are reported in the materials, S2

### Gender-based comparison

Compared to their male counterparts, women were more frequently trainees or surgeons transitioning to independent practice, with a significant difference noted (p = 0.003). Mentoring for women was less focused on surgical skills (57.1 vs. 73.8%), although this difference was not statistically significant (p = 0.11).

### Regional comparison

Acknowledging that our data were biased by an overrepresentation of southern European respondents (reflecting the peer network of the yESTES authors), we identified a prevalence of trauma/emergency general surgeons from southern Europe, 51.8%, and eastern Europe, 63.6%, and visceral (45.8%) and skeletal trauma (45.8%) surgeons from western-central Europe, p < 0.001.

### Career stage-based comparison

Trainees and surgeons in transition to independent practice were less likely to be mentored on surgical skills compared to surgeons in established practice, respectively 54.1 vs. 66.3 vs. 83.1%, p = 0.012. Indeed, early career surgeons and trainees felt that surgical skills were the most important to be addressed within a shoulder-to-shoulder relationship, considering non-technical skill and research/academic skill less important. Furthermore, career progression was identified as an additional priority, especially from surgeons in transition to independent practice, p = 0.05.

### Specialty-based comparison

NTS were identified as essential surgical skills, especially by visceral and EGS surgeons, compared to skeletal trauma surgeons, p = 0.043. Visceral trauma surgeons reported a gap in NTS mentoring compared to surgeons focused on EGS or skeletal trauma, respectively 7.1 vs. 35.2 and 38.5%, p < 0.001. The results of the comparisons are depicted in the supplementary materials S3.

## Discussion

Surgical skill, a sum of acquired knowledge, deliberate practice, and experience, is associated with improved patient outcomes [10]. This skill is best acquired with structured constructive support. [11] This yESTES-led inquiry delved into mentorship accessibility across Europe, canvassing insights from 123 visceral trauma, skeletal trauma, and emergency general surgeons, ESTES members, in 20 countries. Most respondents confirmed mentorship encounters, albeit with mixed experiences and scant formal supports. The study spotlighted particularly at-risk groups. Trainees, particularly female surgeons, are disadvantaged by mentorship voids. Key areas for enhancement emerged, emphasizing NTS mentorship and surgical societie’s potential roles in mending the mentorship breach, proposing both short and long-term plans for surgical trainees and those new to practice (Table 2).

**Table 2:** Summary of study significance

Significance of the study (Why mentorship matters)	
Mentorship dynamics	We observed that 74% of the survey respondents recognized the existence of mentorship, predominantly in an informal setting focused largely on surgical training, indicating a lack of formalized mentorship programs
Discrepancy in early career development	Predominantly, seasoned surgeons reported involvement in mentoring, revealing a significant discrepancy in mentorship availability for surgeons at the onset of their careers and for surgical trainees, highlighting a crucial gap in early professional development
Disparity based on gender	Only 28.5% of survey participants were women, reflecting the gender distribution within the society (23%). This demographic was more likely to be trainees or surgeons transitioning to independent practice, suggesting a notable mentorship gap based on gender
Overemphasis on technical skills	The mentorship provided appeared to inadequately cover non-technical skills, particularly among surgeons specializing in visceral trauma. Most respondents deemed such skills crucial for the ideal mentee, revealing a misalignment between mentorship practices and perceived needs
Role of surgical societies in mentorship	We identified a gap in the contribution of surgical societies to mentorship, specifically within the realms of emergency, visceral, and skeletal trauma surgery. ESTES members prioritized interventions such as traveling fellowships and remote mentoring programs, signaling a need for structured initiatives from organizations such as ESTES

Peer guidance, as evidenced by the Association of Surgeons of Great Britain and Ireland’s Early Years Career Consultant Network, is emerging as a novel mentorship model, transcending traditional mentor–mentee dynamics, and offering a mutual support network which can significantly enhance early-career experiences [12, 13]. Young ESTES mirrors this initiative, fostering education and collaboration among Europe’s emergent emergency and trauma surgeons. Leveraging social networks and digital platforms for knowledge exchange has proven invaluable, as initiatives like Surgical Pizza and Behind The Knife [14, 15]. However, the effects of such peer mentorship on career development trauma and emergency surgery require future longitudinal investigation.

Many ESTES survey participants reported mentor interactions, aligning with findings from the UK and Canada, where structured programs have been noted to foster successful mentor–mentee matches [16, 17]. Similar trends were observed in Switzerland, with informally mentored surgeons, particularly men, enjoying enhanced career progression [18]. A stark contrast was observed in a South African survey, highlighting a profound mentorship void despite recognizing its importance [19]. A needs analysis of US surgeons entering practice identified the broad themes of acquiring confidence in independent operating, integrating into a new organizational culture, dealing with bureaucracy, and balancing work and life. This work found an association between the absence of a mentoring system and adverse patient outcomes, stunted career development and higher rates of attrition from surgical practice [20]. Our work, aligned with findings elsewhere, underscores the urgent need for structured support focusing on both technical and non-technical skills during crucial career transitions. Specifically, and perhaps counterintuitively, both surgeons in transition to independent practice and trainees reported a lack of surgical mentoring compared to surgeons in established practice, reflecting the loss of a contemporary culture of formal mentoring.

The gender gap in surgery remains stark; this is reflected by underrepresentation at all levels within ESTES, in common with other surgical societies [21–23]]. Few female senior role models compounds the lack of mentorship opportunities for female early career surgeons and those in training. A recent scoping review showed that heavy workloads, ineffective mentorship and unclear career pathways represent further obstacles for women in surgery during the early career phase [24]. Furthermore, gender-based harassment whether personally directed or not, exerts a deleterious impact on the desirability of surgical training. The experience of our female ESTES members aligns with the existing literature, underscoring the necessity of bolstered support for aspiring female surgeons [25] and highlighting the obstacles and barriers related to gender-biased discrimination and the interplay of career with additional environmental and societal demands [25]. Increasing visibility and representation of successful female trauma surgeons through conferences, workshops, and medical professional societies can be inspirational. Moreover, providing equal opportunities for training and career advancement, addressing implicit biases in selection processes, and promoting work-life balance initiatives are key steps towards attracting and retaining more women in trauma and emergency surgery [26]. Several societies, such as Women In Surgery (WIS) and the American Medical Women’s Association (AMWA) offer academic and career progression support to female final year medical students through structured remote mentoring programs [27–30].

Traumatologists and emergency general surgeons render complex life-saving care to critically ill patients, often in a time-compressed fashion. Non-technical skills (NTS) augment a surgeon’s ability to communicate effectively, coordinate team members and manage scenarios where partial information compounds high degrees of hazard [28, 31–33]. While these attributes were recognized by our survey respondents, our findings reveal a discrepancy between the perceived importance of these skills and their prioritization within mentorship relationships. This gap highlights an opportunity for targeted educational initiatives from surgical societies.

### Identified opportunities for ESTES

**Fig. 3:**
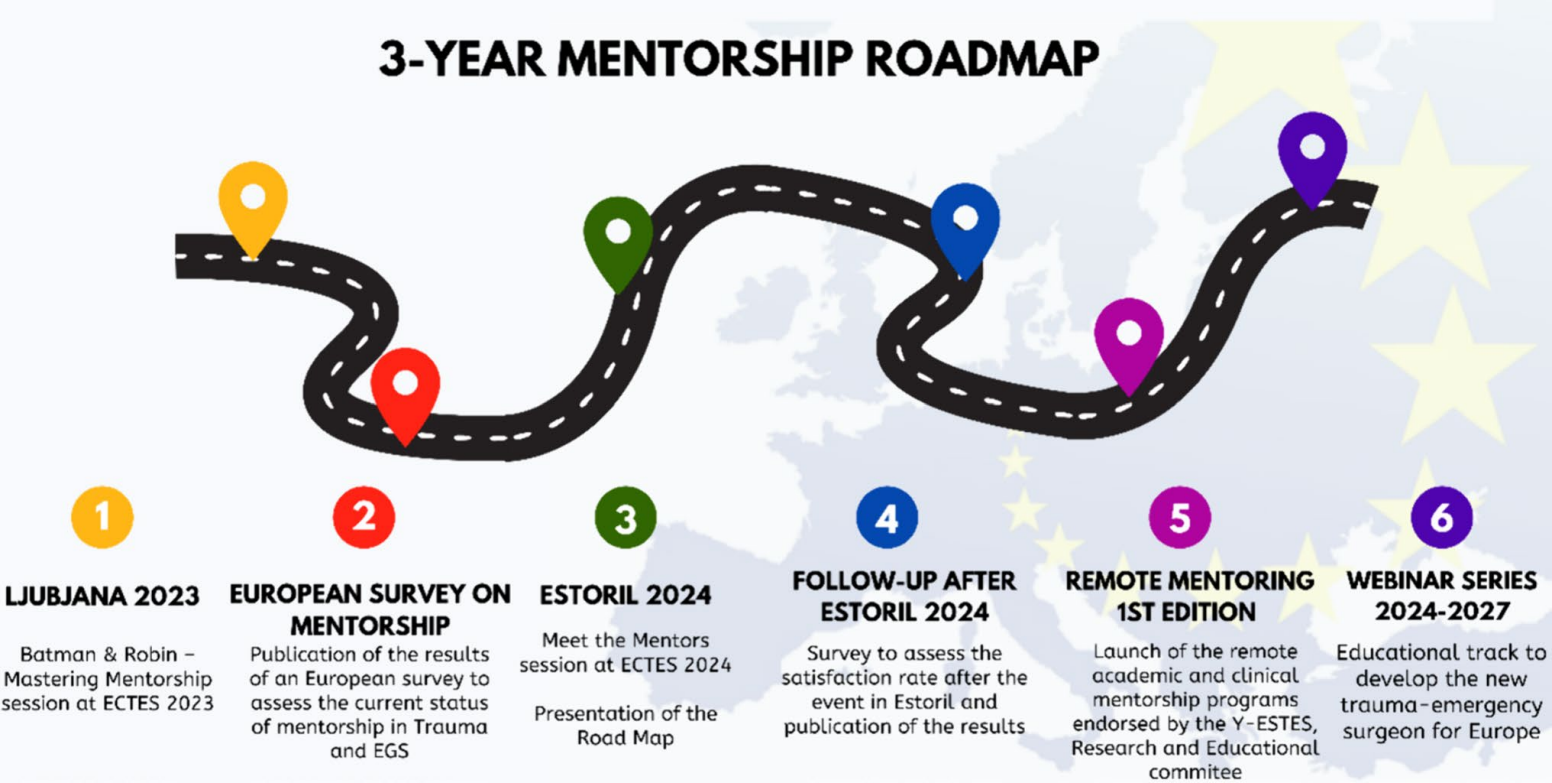
Roadmap of the proposed initiatives on mentorship endorsed by ESTES within the three years from ECTES in Ljubljana 2023. Created by SPB Cioffi, MD (yESTES)

The mentorship landscape in European traumatology and emergency surgery needs a strategic overhaul. Medical professional societies, such as ESTES, occupy a unique space where targeted support and innovative programs lay a path to a more inclusive, skilled, and supported European surgical community. Opportunities include recognition and closure of the curriculum and mentorship gaps in career development sponsorship, research methodology and non-technical skill acquisition support and networking and research collaboration opportunities in addition to the traditional peri-congress technical skill courses offered by such societies. To this end the yESTES, along with the Research and Educational committees, propose a strategy to bridge these gaps (Fig. 3).

Surgical societies, especially those with a research committee, can create an environment in which international multicenter collaborative studies can be conducted [34]. Aspiring academic surgeons should be guided by experienced surgical research mentors in the refinement of a research question and the complex task of writing a scientific paper [14, 26]]. Individual shoulder-to-shoulder mentorship can be supplemented by the creation of a structured European research methods curriculum in emergency and trauma surgery, coupled to the possibility for aspiring surgeon-scientists to apply for research scholarships to fully embrace impactful research [35]. Brief intensive traveling clinical fellowship programs allow trainees and surgeons new to practice to visit host institutions for 1–3 months to acquire a particular skill and to broaden their peer and mentor networks [36–38]]. Funding concerns can be mitigated with strategic planning and securing grants and sponsorship support from surgical societies (such as ESTES), institutional grants, and possible sponsorships from industry partners. Such experiences have unique features that may overcome the logistical shortcomings of the different working environments in Europe, in which surgeons may not always afford to leave their workplace for an entire year, allowing them to create unique relationships with hosting surgeons and expand their support networks.

Remote career mentorship programs may also provide novel opportunities for clinical and academic development, offering flexibility and overcoming economic and cultural barriers [35, 39]]. Furthermore, extramural remote clinical mentorship programs are readily deployed, involving mentees in a productive networked relationship that supports career development, through asymmetric case-based discussion of complex surgical decision making [40]. While there is limited possibility to coach the mentee on surgical technical skills, opportunities to refine skill in perioperative management of complex cases abounds. This kind of extramural relationship could expose new surgeons to alternative clinical approaches and destigmatize requesting technical and psychologic support during the transition to independent practice [41]. Drawing from successful American models, this strategy aims to match mentors and mentees based on specific strengths and weaknesses, leading to significant satisfaction and performance improvements in identified areas [42, 43]].

One of the new yESTES initiatives is a ‘Meet the Mentors’ session at the annual society Congress, modeled on successful experiences in medical professional societies in the United States [44]. Aspiring surgeons have the chance to spend 1 h having short informal chats with senior experts in the field, exploring disparate areas of interest from work/life balance to surgical coaching, getting the unique chance to create a link with mentors as a springboard for longitudinal mentoring relationships. When the 19 mentee participants were asked to identify their priority areas for discussion with the mentors in preparation for this session, career development (15, 78.9%) was deemed as important as advice on refinement of surgical skills (15, 78.9%). Navigating the transition to independent practice (9, 47.4%), acquisition of research skills (8, 42.1%), work-life balance (6, 31.6%) and advice on how to become a good mentor (4, 21.1%) were identified as further mentee priorities.

### Study strengths and limitations

Our survey is the first European exploration of mentorship experiences among surgeons dedicated to trauma care (visceral or skeletal) and emergency general surgery. Through capturing the opinions of a broad cohort of ESTES members, we hope it is representative of the current population of surgeons dedicated to the care of critically ill surgical and trauma patients in Europe. The percentage of women among the survey respondents (28%) mirrors their representation within the society (23%) and is broadly representative. There is the potential for a responder bias, and future work could embrace external validation of our instrument in other settings. A limiting factor could be the online nature of the survey, as ‘email burnout’ has been reported as a negative contributor to survey response rates in a recent systematic review in Annals of Surgery. Thus, a non-response bias cannot be excluded in this study [45]. We received 123 responses from 413 polled members over the two-month survey period, which corresponds to a response rate of approximately 30%. This is within the typical range for email-promulgated online surveys, where response rates can vary from 4 to 68%, with a median of 25%. Factors influencing response rates include research design, participant motivation, and researcher motivation [45].

## Conclusions

Our survey of the European trauma and emergency surgery training landscape reveals gaps and disparities within current mentorship practices that particularly affect early-career and female surgeons. Informal, technical skill-based mentorship encounters predominate, while more structured, inclusive programs that encompass technical expertise, decision-making, academic pursuit and non-technical skills are lacking. Surgical societies, particularly ESTES, are poised to play a transformative role in bridging these gaps, fostering a nurturing environment that promotes a balanced and comprehensive mentorship experience. Through initiatives such as formal remote research curricula, mentorship programs, and traveling fellowships, there is a unique opportunity to redefine and enrich the mentorship paradigm, ultimately enhancing surgical performance and patient outcomes across Europe. This study not only identifies critical areas for intervention but also sets the stage for a more inclusive, supportive, and well-rounded surgical community, underpinning the need for ongoing research into the impacts of diverse mentorship structures on both surgeons and patient care.

## Supplementary Information

Below is the link to the electronic supplementary material.

Supplementary file1 (PDF 37 KB)

Supplementary file2 (PDF 204 KB)

Supplementary file3 (PDF 273 KB)

## Data Availability

Data is provided within the manuscript or supplementary information files.
