# Supplementary material for: Identifying and addressing mentorship gaps in European trauma and emergency surgical training. Results from the Young European Society of Trauma and Emergency Surgery (yESTES) mentorship survey
Source: Eur J Trauma Emerg Surg. 2024 Aug 9;50(5):2539–49. doi: 10.1007/s00068-024-02610-y (PMC11599355; doi:10.1007/s00068-024-02610-y)
Supplement: Supplementary file 1 — Supplementary file1 (PDF 37 KB) [file 68_2024_2610_MOESM1_ESM.pdf]

Structure of the questionnaire sent to ESTES members

Part 1. Demographics

Question 1.
Which is your nationality?
Open question with short written answer

Question 2.
Which is your age?
Open question with short written answer

Question 3.
Which is your gender?
Female
Male
Other

Question 4
Which is your current position?
Resident
Attending surgeon < 40 years
Attending surgeon > 40 years
Chief/Professor

Question 5
Which is your specialty
Emergency General Surgery
Trauma Surgery
Orthopedic/trauma surgery
Surgical critical care

Question 6
In which type of hospital do you work?
Large referral academic hospital
Rural community hospital
Urban community hospital

Question 7
Is the training the same regardless of the hospital and the surgical unit?
Yes
No

<u>Part 2. Mentorship experience</u>

Question 8
Have you ever had a mentor?
Yes
No

Question 9
Have you had one comprehensive mentor?
Yes
No

Question 10
Have you had different mentors on different rotations during your residency?
Yes
No

Question 11
If yes, at which stage of your career did you first meet your mentor?
Medical student
Resident
Fellow
Attending surgeon
Professor

Question 12
In which context did you meet her/him?
Academic context
Workplace
Surgical Society meeting
Surgical courses
Dedicated programs

Question 13
In which field were you mentored?
Surgical
Clinical
Research/academic
Non-technical skills
Preparedness
Resiliency

Question 14
When non mentored, did you manage to get growth opportunities?

Yes
No

Question 15
If yes, which tools and opportunities did you use?
Passive learning
Surgical videos
Studying
Surgical courses

<u>Part 3. Perceptions of mentorship</u>
Question 16
Which skills are essential to a mentee to deserve to be mentored and take advantage of the relationship?
Technical skills
Non-technical skills
Academic skills

Question 17
Should mentorship be guaranteed to each surgical trainee?
Yes
No

Question 18
Which is the best moment of the career to find a mentor?
Medical school
Residency
Fellowship
Workplace
Does not matter

Question 19
Do you believe that a mentor-mentee relationship should be based on a strong personal connection?
Yes
No

Question 20
Do you believe that a mentor should work in the same hospital of the mentee?
Yes
No

Question 21

Which area do you believe would benefit more from a shoulder-to-shoulder mentorship?
Surgical skills
Academic scientific skills
Non-technical skills
Networking
Career progression
Coaching skills
Life work balance
Follow evidence based practices

Question 22
Do you believe that a remote mentorship could be effective?
Yes
No

Question 23
Which area do you believe would benefit more from a remote mentorship?
Surgical skills
Academic scientific skills
Non-technical skills
Networking
Career progression
Coaching skills
Life work balance
Follow evidence based practices

Question 22
In terms of clinical and surgical skills: how do you or did you take advantage of your mentor?
Preoperative decision making
Intraoperative decision making
Clinical management of complications
Mental rehearsal after a complex case or a complication
Clinical mentorship of youngers

Question 24
How do you mentor opinions impact your daily practice?
I promptly change my practice If I get a different insight/opinion
I carefully consider the opinion in my decision making process and then I make my call

Question 25

Do you believe that mental wellbeing of young trauma and emergency surgeons, especially related to tough clinical scenarios and life-work balance, can be positively influenced by having a mentor?
Yes
No

Part 4. Research gaps and future interventions

Question 26
Do you feel that this area of study should be expanded in Europe?
Yes
No

Question 27
Do you believe that the mentor-mentee relationship can be oriented by institutional programs?
Yes
No

Question 28
Should universities be in charge of pairing mentors and mentees during surgical career?
Yes, for medical students
Yes, for residents
Yes, for fellows
Yes, for attending
Yes, regardless of the status
No

Question 29
Which of the following initiatives you believe would be more effective to implement mentorship in the field of emergency and trauma surgery in Europe?
Remote clinical mentorship program
Remote academic/scientific mentoring program
Traveling clinical fellowship
Dedicated research fellowship
Surgical hands-on courses
Summer school in medical writing
Non-technical skills courses
