# Supplementary material for: Identifying and addressing mentorship gaps in European trauma and emergency surgical training. Results from the Young European Society of Trauma and Emergency Surgery (yESTES) mentorship survey
Source: Eur J Trauma Emerg Surg. 2024 Aug 9;50(5):2539–49. doi: 10.1007/s00068-024-02610-y (PMC11599355; doi:10.1007/s00068-024-02610-y)
Supplement: Supplementary file 2 — Supplementary file2 (PDF 204 KB) [file 68_2024_2610_MOESM2_ESM.pdf]

**Variable**	**Mean (SD)**<br>**Count (%)**	**95% CI**	**Min**<br>**Max**	**Q1**<br>**Q3**	**Median**
**Type of Nation**					
Southern Europe	85 (69.1)	60.9%<br>77.3%			
Western-Central Europe	24 (19.5)	12.5%<br>26.5%			
Northern Europe	11 (8.9)	3.9%<br>14.0%			
Eastern Europe	3 (2.4)	0%<br>5.2%			
**Gender**					
male	88 (71.5)	63.6%<br>79.5%			
female	35 (28.5)	20.5%<br>36.4%			
**Age**	41.11 (11.19)	39.11<br>43.1	26<br>68	31.5<br>47.5	39
**Position**					
Surgeon in established practice	56 (45.5)	36.7%			

Variable	Mean (SD)<br>Count (%)	95% CI	Min<br>Max	Q1<br>Q3	Median
		54.3%			
Resident	37 (30.1)	22.0%<br>38.2%			
Surgeon in transition to independent practice	30 (24.4)	16.8%<br>32.0%			
**Speciality**					
Visceral Trauma	56 (45.5)	36.7%<br>54.3%			
EGS	54 (43.9)	35.1%<br>52.7%			
Skeletal trauma	13 (10.6)	5.1%<br>16.0%			
**Type of hospital in which you currently work**					
large referral academic hospital	88 (71.5)	63.6%<br>79.5%			
urban community	29 (23.6)	16.1%<br>31.1%			

Variable	Mean (SD)<br>Count (%)	95% CI	Min<br>Max	Q1<br>Q3	Median
rural community	6 (4.9)	1.1%<br>8.7%			
**Have you ever had a mentor?**					
Yes	92 (74.8)	67.1%<br>82.5%			
No	31 (25.2)	17.5%<br>32.9%			
**Have you had one comprehensive mentor?**					
No	62 (53.4)	44.4%<br>62.5%			
Yes	54 (46.6)	37.5%<br>55.6%			
**Have you had different mentors on different rotations during your residency?**					
Yes	80 (69.0)	60.5%<br>77.4%			

Variable	Mean (SD)<br>Count (%)	95% CI	Min<br>Max	Q1<br>Q3	Median
No	36 (31.0)	22.6%<br>39.5%			
**At which stage of your career did you first meet your mentor?**					
Resident	54 (60.0)	49.9%<br>70.1%			
Medical Student	22 (24.4)	15.6%<br>33.3%			
Attending Surgeon	7 (7.8)	2.2%<br>13.3%			
Fellow	5 (5.6)	0.8%<br>10.3%			
Professor	2 (2.2)	0%<br>5.3%			
**In which context you met your mentor?**					
workplace	60 (62.5)	52.8%<br>72.2%			

Variable	Mean (SD)<br>Count (%)	95% CI	Min<br>Max	Q1<br>Q3	Median
academic context	30 (31.2)	22.0%<br>40.5%			
surgical course	4 (4.2)	0.2%<br>8.2%			
dedicated pairing programs	2 (2.1)	0%<br>4.9%			
**Did you receive surgical mentoring?**					
Yes	85 (68.5)	60.4%<br>76.7%			
No	39 (31.5)	23.3%<br>39.6%			
**Did you receive clinical mentoring?**					
No	78 (62.9)	54.4%<br>71.4%			
Yes	46 (37.1)	28.6%<br>45.6%			

Variable	Mean (SD)<br>Count (%)	95% CI	Min<br>Max	Q1<br>Q3	Median
**Were you mentored on research?**					
No	80 (64.5)	56.1%<br>72.9%			
Yes	44 (35.5)	27.1%<br>43.9%			
**Were you mentored on non-technical skills?**					
No	96 (77.4)	70.1%<br>84.8%			
Yes	28 (22.6)	15.2%<br>29.9%			
**Which skills are essential to a mentee to deserve to be mentored and take advantage of the relationship?**					
non-technical skills (trustworthiness, committment)	64 (60.4)	51.1%<br>69.7%			

Variable	Mean (SD)<br>Count (%)	95% CI	Min<br>Max	Q1<br>Q3	Median
technical skills	36 (34.0)	24.9%<br>43.0%			
academic skills	6 (5.7)	1.3%<br>10.1%			
**Which is the best moment of the career to find a mentor?**					
residency	80 (74.8)	66.5%<br>83.0%			
medical school	12 (11.2)	5.2%<br>17.2%			
it does not matter	10 (9.3)	3.8%<br>14.9%			
fellowship	5 (4.7)	0.7%<br>8.7%			
**Do you believe that the mentor-mentee relationship should necessarily be based on a strong personal connection?**					
Yes	69 (64.5)	55.4%			

Variable	Mean (SD)<br>Count (%)	95% CI	Min<br>Max	Q1<br>Q3	Median
		73.6%			
No	38 (35.5)	26.4%<br>44.6%			
**Do you believe that a mentor should work in the same hospital of the mentee?**					
Yes	77 (72.0)	63.5%<br>80.5%			
No	30 (28.0)	19.5%<br>36.5%			
**Which area do you believe would benefit more from a shoulder-to-shoulder mentorship?**					
surgical skills	68 (63.0)	53.9%<br>72.1%			
career progression	15 (13.9)	7.4%<br>20.4%			
non-technical skills	11 (10.2)	4.5%			

Variable	Mean (SD)<br>Count (%)	95% CI	Min<br>Max	Q1<br>Q3	Median
		15.9%			
academic/scientific skills	7 (6.5)	1.8%<br>11.1%			
coaching skills to become a mentor myself	3 (2.8)	0%<br>5.9%			
follow evidence-based practices	2 (1.9)	0%<br>4.4%			
life-work balance	1 (0.9)	0%<br>2.7%			
networking	1 (0.9)	0%<br>2.7%			
**At which moment shoulder to shoulder mentorship would work best?**					
residency	91 (84.3)	77.4%<br>91.1%			
fellowhip	7 (6.5)	1.8%<br>11.1%			
medical student	7 (6.5)	1.8%			

Variable	Mean (SD)<br>Count (%)	95% CI	Min<br>Max	Q1<br>Q3	Median
		11.1%			
attending surgeon	3 (2.8)	0%<br>5.9%			
**Do you believe that a remote mentorship could be effective?**					
Yes	62 (59.0)	49.6%<br>68.5%			
No	43 (41.0)	31.5%<br>50.4%			
**Which area you believe would benefit more from remote mentorship?**					
academic/scientific skills	45 (42.1)	32.7%<br>51.4%			
networking	17 (15.9)	9.0%<br>22.8%			
career progression	15 (14.0)	7.4%<br>20.6%			

Variable	Mean (SD)<br>Count (%)	95% CI	Min<br>Max	Q1<br>Q3	Median
non-technical skills	14 (13.1)	6.7%<br>19.5%			
surgical skills	7 (6.5)	1.9%<br>11.2%			
follow evidence-based practices	4 (3.7)	0.1%<br>7.3%			
work-life balance	3 (2.8)	0%<br>5.9%			
coaching skills to become a mentor myself	2 (1.9)	0%<br>4.4%			
**In terms of clinical and surgical skills: how do you or did you take advantage of your mentor?**					
intraoperative decision making	30 (30.0)	21.0%<br>39.0%			
preoperative surgical decision making	27 (27.0)	18.3%<br>35.7%			
clinical management of complications	17 (17.0)	9.6%<br>24.4%			

Variable	Mean (SD)<br>Count (%)	95% CI	Min<br>Max	Q1<br>Q3	Median
mental rehearsal after a complex case or a complication	14 (14.0)	7.2%<br>20.8%			
clinical mentorship of younger surgeons/residents/students	12 (12.0)	5.6%<br>18.4%			
**How do your mentor opinions impact your daily practice?**					
I carefully consider its opinion in my decision making process and then I make my call	78 (81.2)	73.4%<br>89.1%			
I promptly change my practice if I get a different insight/opinion from my mentor	18 (18.8)	10.9%<br>26.6%			
**Do you believe that mental wellbeing of young trauma and emergency surgeons, especially related to tough clinical scenarios and life-work balance, can be**					

Variable	Mean (SD)<br>Count (%)	95% CI	Min<br>Max	Q1<br>Q3	Median
**positively influenced by having a mentor?**					
Yes	95 (96.0)	92.1%<br>99.8%			
No	4 (4.0)	0.2%<br>7.9%			
**Do you feel this area of study should be expanded in Europe?**					
Yes	93 (93.9)	89.2%<br>98.6%			
No	6 (6.1)	1.4%<br>10.8%			
**Do you believe that the mentor-mentee relationship can be oriented by institutional programs?**					
Yes	93 (93.9)	89.2%<br>98.6%			

Variable	Mean (SD)<br>Count (%)	95% CI	Min<br>Max	Q1<br>Q3	Median
No	6 (6.1)	1.4%<br>10.8%			
**Should universities be in charge of pairing mentors and mentees during surgical career?**					
Yes for surgery residents	55 (55.6)	45.8%<br>65.3%			
Yes regardless of the status	18 (18.2)	10.6%<br>25.8%			
No	14 (14.1)	7.3%<br>21.0%			
Yes for medical students	8 (8.1)	2.7%<br>13.4%			
Yes for surgical fellows	2 (2.0)	0%<br>4.8%			
Yes for young attending	2 (2.0)	0%<br>4.8%			
**Should scientific societies be in charge of pairing mentors**					

Variable	Mean (SD)<br>Count (%)	95% CI	Min<br>Max	Q1<br>Q3	Median
**and mentees during surgical career?**					
Yes for surgery residents	34 (34.7)	25.3%<br>44.1%			
No	21 (21.4)	13.3%<br>29.6%			
Yes regardless of the status	20 (20.4)	12.4%<br>28.4%			
Yes for surgical fellows	18 (18.4)	10.7%<br>26.0%			
Yes for young attending	5 (5.1)	0.7%<br>9.5%			

S2. Results of the survey.
