# Supplementary material for: Identifying and addressing mentorship gaps in European trauma and emergency surgical training. Results from the Young European Society of Trauma and Emergency Surgery (yESTES) mentorship survey
Source: Eur J Trauma Emerg Surg. 2024 Aug 9;50(5):2539–49. doi: 10.1007/s00068-024-02610-y (PMC11599355; doi:10.1007/s00068-024-02610-y)
Supplement: Supplementary file 3 — Supplementary file3 (PDF 273 KB) [file 68_2024_2610_MOESM3_ESM.pdf]

Supplementary materials 3, S3.

Group comparisons.

S3.1 Group comparison according to gender

Variable	male<br>N = 88	female<br>N = 35	p-Value
**Type of Nation**			0.856
Eastern Europe	3 (3.41%)	0 (0.0%)	
Northern Europe	8 (9.09%)	3 (8.57%)	
Southern Europe	59 (67.05%)	26 (74.29%)	
Western-Central Europe	18 (20.45%)	6 (17.14%)	
	N = 88	N = 35	
**Age**	**43.31 (± 11.38)<br>Range: (27.0 ; 68.0)<br>N = 88**	**35.57 (± 8.58)<br>Range: (26.0 ; 65.0)<br>N = 35**	**<0.001**
**Position**			**0.003**
Resident	**20 (22.73%)**	**17 (48.57%)**	
Surgeon in established practice	**48 (54.55%)**	**8 (22.86%)**	
Surgeon in transition to independent practice	**20 (22.73%)**	**10 (28.57%)**	
	**N = 88**	**N = 35**	
**Speciality**			0.862
EGS	39 (44.32%)	15 (42.86%)	
Skeletal trauma	10 (11.36%)	3 (8.57%)	
Visceral Trauma	39 (44.32%)	17 (48.57%)	
	N = 88	N = 35	
**Type of hospital in which you currently work**			0.879
large referral academic hospital	63 (71.59%)	25 (71.43%)	
rural community	5 (5.68%)	1 (2.86%)	
urban community	20 (22.73%)	9 (25.71%)	
	N = 88	N = 35	
**Have you ever had a mentor?**			0.36
Yes	68 (77.27%)	24 (68.57%)	
No	20 (22.73%)	11 (31.43%)	
	N = 88	N = 35	
**Have you had one comprehensive mentor?**			0.238
Yes	42 (50.6%)	12 (36.36%)	
No	41 (49.4%)	21 (63.64%)	
	N = 83	N = 33	
**Have you had different mentors on different rotations during your residency?**			0.147
Yes	61 (73.49%)	19 (57.58%)	
No	22 (26.51%)	14 (42.42%)	
	N = 83	N = 33	
**At which stage of your career did you first meet your mentor?**			0.421
Trained Surgeon	4 (5.97%)	3 (13.04%)	
Fellow	4 (5.97%)	1 (4.35%)	
Medical Student	19 (28.36%)	3 (13.04%)	
Professor	2 (2.99%)	0 (0.0%)	
Resident	38 (56.72%)	16 (69.57%)	
	N = 67	N = 23	
**In which context you met your mentor?**			0.086
academic context	24 (33.8%)	6 (24.0%)	
dedicated pairing programs	0 (0.0%)	2 (8.0%)	
surgical course	4 (5.63%)	0 (0.0%)	
workplace	43 (60.56%)	17 (68.0%)	
	N = 71	N = 25	
**Did you receive surgical mentoring?**			0.111
Yes	65 (73.86%)	20 (57.14%)	
No	23 (26.14%)	15 (42.86%)	

	N = 88	N = 35	
**Did you receive clinical mentoring?**			0.512
Yes	35 (39.77%)	11 (31.43%)	
No	53 (60.23%)	24 (68.57%)	
	N = 88	N = 35	
**Were you mentored on research?**			0.993
Yes	32 (36.36%)	12 (34.29%)	
No	56 (63.64%)	23 (65.71%)	
	N = 88	N = 35	
**Were you mentored on non-technical skills?**			>0.999
Yes	20 (22.73%)	8 (22.86%)	
No	68 (77.27%)	27 (77.14%)	
	N = 88	N = 35	
**Which skills are essential to a mentee to deserve to be mentored and take advantage of the relationship?**			0.345
academic skills	6 (7.79%)	0 (0.0%)	
non-technical skills	46 (59.74%)	18 (62.07%)	
technical skills	25 (32.47%)	11 (37.93%)	
	N = 77	N = 29	
**Which is the best moment of the career to find a mentor?**			0.286
fellowship	5 (6.49%)	0 (0.0%)	
it does not matter	9 (11.69%)	1 (3.33%)	
medical school	8 (10.39%)	4 (13.33%)	
residency	55 (71.43%)	25 (83.33%)	
	N = 77	N = 30	
**Do you believe that the mentor-mentee relationship should necessarily be based on a strong personal connection?**			0.333
Yes	47 (61.04%)	22 (73.33%)	
No	30 (38.96%)	8 (26.67%)	
	N = 77	N = 30	
**Do you believe that a mentor should work in the same hospital of the mentee?**			0.478
Yes	57 (74.03%)	20 (66.67%)	
No	20 (25.97%)	10 (33.33%)	
	N = 77	N = 30	
**Which area do you believe would benefit more from a shoulder-to-shoulder mentorship?**	7 (8.97%)	0 (0.0%)	0.443
academic/scientific skills	8 (10.26%)	7 (23.33%)	
career progression	2 (2.56%)	1 (3.33%)	
mentoring skill	2 (2.56%)	0 (0.0%)	
follow evidence-based practices	1 (1.28%)	0 (0.0%)	
life-work balance	1 (1.28%)	0 (0.0%)	
networking	8 (10.26%)	3 (10.0%)	
non-technical skills	49 (62.82%)	19 (63.33%)	
surgical skills	N = 78	N = 30	
**At which moment shoulder to shoulder mentorship would work best?**			0.83
attending surgeon	3 (3.85%)	0 (0.0%)	
fellowhip	5 (6.41%)	2 (6.67%)	
medical student	6 (7.69%)	1 (3.33%)	
residency	64 (82.05%)	27 (90.0%)	
	N = 78	N = 30	
**Do you believe that a remote mentorship could be effective?**	44 (58.67%)	18 (60.0%)	>0.999
Yes	31 (41.33%)	12 (40.0%)	
No	N = 75	N = 30	
**Which area you believe would benefit more from remote mentorship?**			0.533
academic/scientific skills	32 (41.56%)	13 (43.33%)	
career progression	9 (11.69%)	6 (20.0%)	
mentoring skills	1 (1.3%)	1 (3.33%)	
follow evidence-based practices	4 (5.19%)	0 (0.0%)	
networking	12 (15.58%)	5 (16.67%)	
non-technical skills	12 (15.58%)	2 (6.67%)	

surgical skills	4 (5.19%)	3 (10.0%)	
work-life balance	3 (3.9%)	0 (0.0%)	
	N = 77	N = 30	
**In terms of clinical and surgical skills: how do you or did you take advantage of your mentor?**			0.801
clinical management of complications	13 (18.06%)	4 (14.29%)	
be a mentors myself	7 (9.72%)	5 (17.86%)	
intraoperative decision making	23 (31.94%)	7 (25.0%)	
mental rehearsal	10 (13.89%)	4 (14.29%)	
preoperative surgical decision making	19 (26.39%)	8 (28.57%)	
	N = 72	N = 28	
**How do your mentor opinions impact your daily practice?**			0.085
I carefully consider its opinion in my decision making process and then I make my call	52 (76.47%)	26 (92.86%)	
I promptly change my practice if I get a different insight/opinion from my mentor	16 (23.53%)	2 (7.14%)	
	N = 68	N = 28	
**Do you believe that mental wellbeing of young trauma and emergency surgeons, especially related to tough clinical scenarios and life-work balance, can be positively influenced by having a mentor?**			>0.999
Yes	67 (95.71%)	28 (96.55%)	
No	3 (4.29%)	1 (3.45%)	
	N = 70	N = 29	
**Do you feel this area of study should be expanded in Europe?**			0.176
Yes	64 (91.43%)	29 (100.0%)	
No	6 (8.57%)	0 (0.0%)	
	N = 70	N = 29	
**Do you believe that the mentor-mentee relationship can be oriented by institutional programs?**			0.176
Yes	64 (91.43%)	29 (100.0%)	
No	6 (8.57%)	0 (0.0%)	
	N = 70	N = 29	
**Should universities be in charge of pairing mentors and mentees during surgical career?**			**0.023**
No	13 (18.57%)	1 (3.45%)	
Yes for medical students	2 (2.86%)	6 (20.69%)	
Yes for surgery residents	39 (55.71%)	16 (55.17%)	
Yes for surgical fellows	2 (2.86%)	0 (0.0%)	
Yes for young attending	2 (2.86%)	0 (0.0%)	
Yes regardless of the status	12 (17.14%)	6 (20.69%)	
	N = 70	N = 29	
**Should scientific societies be in charge of pairing mentors and mentees during surgical career?**			0.09
No	15 (21.74%)	6 (20.69%)	
Yes for surgery residents	22 (31.88%)	12 (41.38%)	
Yes for surgical fellows	17 (24.64%)	1 (3.45%)	
Yes for young attending	2 (2.9%)	3 (10.34%)	
Yes regardless of the status	13 (18.84%)	7 (24.14%)	
	N = 69	N = 29	

S3.2 Group comparison according to the career stage.

	Surgeon in established practice N = 56	Resident N = 37	Surgeon in transition to independent practice N = 30	p-Value
**Type of Nation**				**0.003**
Eastern Europe	2 (3.57%)	0 (0.0%)	1 (3.33%)	
Northern Europe	8 (14.29%)	3 (8.11%)	0 (0.0%)	
Southern Europe	29 (51.79%)	29 (78.38%)	27 (90.0%)	
Western-Central Europe	17 (30.36%)	5 (13.51%)	2 (6.67%)	
	N = 56	N = 37	N = 30	
**Age**	50.29 (± 8.05)	29.84 (± 2.25)	37.87 (± 7.87)	**<0.001**
	Range: (40.0 ; 68.0)	Range: (26.0 ; 36.0)	Range: (32.0 ; 65.0)	
	N = 56	N = 37	N = 30	
**Gender**				**0.003**
Female	8 (14.29%)	17 (45.95%)	10 (33.33%)	
Male	48 (85.71%)	20 (54.05%)	20 (66.67%)	
	N = 56	N = 37	N = 30	
**Speciality**				0.138
EGS	22 (39.29%)	15 (40.54%)	17 (56.67%)	
Skeletal trauma	9 (16.07%)	4 (10.81%)	0 (0.0%)	
Visceral Trauma	25 (44.64%)	18 (48.65%)	13 (43.33%)	
	N = 56	N = 37	N = 30	
**Type of hospital in which you currently work**				0.488
large referral academic	41 (73.21%)	24 (64.86%)	23 (76.67%)	
rural community	1 (1.79%)	3 (8.11%)	2 (6.67%)	
urban community	14 (25.0%)	10 (27.03%)	5 (16.67%)	
	N = 56	N = 37	N = 30	
**Have you ever had a mentor?**				0.168
Yes	46 (82.14%)	24 (64.86%)	22 (73.33%)	
No	10 (17.86%)	13 (35.14%)	8 (26.67%)	
	N = 56	N = 37	N = 30	
**Have you had one comprehensive mentor?**				0.746
Yes	27 (50.0%)	15 (45.45%)	12 (41.38%)	
No	27 (50.0%)	18 (54.55%)	17 (58.62%)	
	N = 54	N = 33	N = 29	
**Have you had different mentors on different rotations during your residency?**				0.347
Yes	40 (74.07%)	23 (69.7%)	17 (58.62%)	
No	14 (25.93%)	10 (30.3%)	12 (41.38%)	
	N = 54	N = 33	N = 29	
**At which stage of your career did you first meet your mentor?**				0.328
Trained Surgeon	4 (8.7%)	2 (8.7%)	1 (4.76%)	
Fellow	1 (2.17%)	1 (4.35%)	3 (14.29%)	
Medical Student	10 (21.74%)	4 (17.39%)	8 (38.1%)	
Professor	2 (4.35%)	0 (0.0%)	0 (0.0%)	
Resident	29 (63.04%)	16 (69.57%)	9 (42.86%)	
	N = 46	N = 23	N = 21	
**In which context you met your mentor?**				0.407
academic context	14 (28.57%)	5 (20.83%)	11 (47.83%)	
dedicated pairing programs	1 (2.04%)	1 (4.17%)	0 (0.0%)	
surgical course	3 (6.12%)	1 (4.17%)	0 (0.0%)	
workplace	31 (63.27%)	17 (70.83%)	12 (52.17%)	
	N = 49	N = 24	N = 23	
**Did you receive surgical mentoring?**				**0.012**
Yes	46 (82.14%)	20 (54.05%)	19 (63.33%)	
No	10 (17.86%)	17 (45.95%)	11 (36.67%)	
	N = 56	N = 37	N = 30	
**Did you receive clinical mentoring?**				0.302
Yes	25 (44.64%)	11 (29.73%)	10 (33.33%)	
No	31 (55.36%)	26 (70.27%)	20 (66.67%)	

	N = 56	N = 37	N = 30	
**Were you mentored on research?**				0.992
Yes	20 (35.71%)	13 (35.14%)	11 (36.67%)	
No	36 (64.29%)	24 (64.86%)	19 (63.33%)	
	N = 56	N = 37	N = 30	
**Were you mentored on non-technical skills?**				0.239
Yes	12 (21.43%)	6 (16.22%)	10 (33.33%)	
No	44 (78.57%)	31 (83.78%)	20 (66.67%)	
	N = 56	N = 37	N = 30	
**Which skills are essential to a mentee to deserve to be mentored and take advantage of the relationship?**				0.135
academic skills	6 (11.76%)	0 (0.0%)	0 (0.0%)	
non-technical skills	31 (60.78%)	16 (57.14%)	17 (62.96%)	
technical skills	14 (27.45%)	12 (42.86%)	10 (37.04%)	
	N = 51	N = 28	N = 27	
**Which is the best moment of the career to find a mentor?**				0.061
fellowship	2 (3.92%)	3 (10.34%)	0 (0.0%)	
it does not matter	5 (9.8%)	2 (6.9%)	3 (11.11%)	
medical school	2 (3.92%)	3 (10.34%)	7 (25.93%)	
residency	42 (82.35%)	21 (72.41%)	17 (62.96%)	
	N = 51	N = 29	N = 27	
**Do you believe that the mentor-mentee relationship should necessarily be based on a strong personal connection?**				0.979
Yes	33 (64.71%)	19 (65.52%)	17 (62.96%)	
No	18 (35.29%)	10 (34.48%)	10 (37.04%)	
	N = 51	N = 29	N = 27	
**Do you believe that a mentor should work in the same hospital of the mentee?**				0.763
Yes	38 (74.51%)	21 (72.41%)	18 (66.67%)	
No	13 (25.49%)	8 (27.59%)	9 (33.33%)	
	N = 51	N = 29	N = 27	
**Which area do you believe would benefit more from a shoulder-to-shoulder mentorship?**				0.057
academic/scientific skills				
career progression	7 (13.46%)	0 (0.0%)	0 (0.0%)	
mentoring skill	6 (11.54%)	3 (10.34%)	6 (22.22%)	
follow evidence-based practices	3 (5.77%)	0 (0.0%)	0 (0.0%)	
life-work balance	1 (1.92%)	0 (0.0%)	1 (3.7%)	
networking	1 (1.92%)	0 (0.0%)	0 (0.0%)	
non-technical skills	0 (0.0%)	1 (3.45%)	0 (0.0%)	
surgical skills	7 (13.46%)	1 (3.45%)	3 (11.11%)	
	27 (51.92%)	24 (82.76%)	17 (62.96%)	
	N = 52	N = 29	N = 27	
**At which moment shoulder to shoulder mentorship would work best?**				0.285
attending surgeon				
fellowhip	2 (3.85%)	0 (0.0%)	1 (3.7%)	
medical student	1 (1.92%)	3 (10.34%)	3 (11.11%)	
residency	2 (3.85%)	3 (10.34%)	2 (7.41%)	
	47 (90.38%)	23 (79.31%)	21 (77.78%)	
	N = 52	N = 29	N = 27	
**Do you believe that a remote mentorship could be effective?**				0.205
Yes	29 (56.86%)	14 (50.0%)	19 (73.08%)	
No	22 (43.14%)	14 (50.0%)	7 (26.92%)	
	N = 51	N = 28	N = 26	
**Which area you believe would benefit more from remote mentorship?**				0.992
academic/scientific skills	20 (38.46%)	14 (50.0%)	11 (40.74%)	
career progression	7 (13.46%)	4 (14.29%)	4 (14.81%)	
mentoring skills	1 (1.92%)	1 (3.57%)	0 (0.0%)	
follow evidence-based practices	2 (3.85%)	1 (3.57%)	1 (3.7%)	
networking	8 (15.38%)	4 (14.29%)	5 (18.52%)	
non-technical skills	8 (15.38%)	2 (7.14%)	4 (14.81%)	
surgical skills	3 (5.77%)	2 (7.14%)	2 (7.41%)	
work-life balance	3 (5.77%)	0 (0.0%)	0 (0.0%)	

	N = 52	N = 28	N = 27	
**In terms of clinical and surgical skills: how do you or did you take advantage of your mentor?**				0.294
clinical management of complications	11 (22.0%)	2 (7.69%)	4 (16.67%)	
be a mentors myself	7 (14.0%)	4 (15.38%)	1 (4.17%)	
intraoperative decision making	15 (30.0%)	8 (30.77%)	7 (29.17%)	
mental rehearsal	9 (18.0%)	3 (11.54%)	2 (8.33%)	
preoperative surgical decision making	8 (16.0%)	9 (34.62%)	10 (41.67%)	
	N = 50	N = 26	N = 24	
**How do your mentor opinions impact your daily practice?**				0.879
I carefully consider its opinion in my decision making process and then I make my call	39 (81.25%)	21 (84.0%)	18 (78.26%)	
I promptly change my practice if I get a different insight/opinion from my mentor	9 (18.75%)	4 (16.0%)	5 (21.74%)	
	N = 48	N = 25	N = 23	
**Do you believe that mental wellbeing of young trauma and emergency surgeons, especially related to tough clinical scenarios and life-work balance, can be positively influenced by having a mentor?**				0.475
Yes	45 (97.83%)	27 (96.43%)	23 (92.0%)	
No	1 (2.17%)	1 (3.57%)	2 (8.0%)	
	N = 46	N = 28	N = 25	
**Do you feel this area of study should be expanded in Europe?**				>0.999
Yes	43 (93.48%)	26 (92.86%)	24 (96.0%)	
No	3 (6.52%)	2 (7.14%)	1 (4.0%)	
	N = 46	N = 28	N = 25	
**Do you believe that the mentor-mentee relationship can be oriented by institutional programs?**				0.273
Yes	45 (97.83%)	25 (89.29%)	23 (92.0%)	
No	1 (2.17%)	3 (10.71%)	2 (8.0%)	
	N = 46	N = 28	N = 25	
**Should universities be in charge of pairing mentors and mentees during surgical career?**				0.886
No	8 (17.39%)	2 (7.14%)	4 (16.0%)	
Yes for medical students	4 (8.7%)	1 (3.57%)	3 (12.0%)	
Yes for surgery residents	24 (52.17%)	18 (64.29%)	13 (52.0%)	
Yes for surgical fellows	1 (2.17%)	1 (3.57%)	0 (0.0%)	
Yes for young attending	1 (2.17%)	0 (0.0%)	1 (4.0%)	
Yes regardless of the status	8 (17.39%)	6 (21.43%)	4 (16.0%)	
	N = 46	N = 28	N = 25	
**Should scientific societies be in charge of pairing mentors and mentees during surgical career?**				0.315
No	10 (21.74%)	2 (7.41%)	9 (36.0%)	
Yes for surgery residents	15 (32.61%)	12 (44.44%)	7 (28.0%)	
Yes for surgical fellows	8 (17.39%)	5 (18.52%)	5 (20.0%)	
Yes for young attending	4 (8.7%)	1 (3.7%)	0 (0.0%)	
Yes regardless of the status	9 (19.57%)	7 (25.93%)	4 (16.0%)	
	N = 46	N = 27	N = 25	

S3.3 Group comparison according to the area of clinical focus

	Visceral Trauma N = 56	EGS N = 54	Skeletal trauma N = 13	p-Value
**Type of Nation**				**<0.001**
Eastern Europe	1 (1.79%)	1 (1.85%)	1 (7.69%)	
Northern Europe	4 (7.14%)	7 (12.96%)	0 (0.0%)	
Southern Europe	40 (71.43%)	44 (81.48%)	1 (7.69%)	
Western-Central Europe	11 (19.64%)	2 (3.7%)	11 (84.62%)	
	N = 56	N = 54	N = 13	
**Age**	41.75 (± 11.9)	39.11 (± 10.02)	46.62 (± 11.3)	0.099
	Range: (27.0 ; 65.0)	Range: (26.0 ; 68.0)	Range: (30.0 ; 60.0)	
	N = 56	N = 54	N = 13	
**Gender**				0.862
Female	17 (30.36%)	15 (27.78%)	3 (23.08%)	
Male	39 (69.64%)	39 (72.22%)	10 (76.92%)	
	N = 56	N = 54	N = 13	
**Position**				0.138
Surgeon in established practice	18 (32.14%)	15 (27.78%)	4 (30.77%)	
Resident	25 (44.64%)	22 (40.74%)	9 (69.23%)	
Surgeon in transition to independent practice	13 (23.21%)	17 (31.48%)	0 (0.0%)	
	N = 56	N = 54	N = 13	
**Type of hospital in which you currently work**				0.404
large referral academic	39 (69.64%)	41 (75.93%)	8 (61.54%)	
rural community	2 (3.57%)	2 (3.7%)	2 (15.38%)	
urban community	15 (26.79%)	11 (20.37%)	3 (23.08%)	
	N = 56	N = 54	N = 13	
**Have you ever had a mentor?**				0.595
Yes	40 (71.43%)	41 (75.93%)	11 (84.62%)	
No	16 (28.57%)	13 (24.07%)	2 (15.38%)	
	N = 56	N = 54	N = 13	
**Have you had one comprehensive mentor?**				0.277
Yes	28 (54.9%)	21 (40.38%)	5 (38.46%)	
No	23 (45.1%)	31 (59.62%)	8 (61.54%)	
	N = 51	N = 52	N = 13	
**Have you had different mentors on different rotations during your residency?**				0.684
Yes	36 (70.59%)	34 (65.38%)	10 (76.92%)	
No	15 (29.41%)	18 (34.62%)	3 (23.08%)	
	N = 51	N = 52	N = 13	
**At which stage of your career did you first meet your mentor?**				0.212
Trained Surgeon	2 (5.0%)	4 (10.26%)	1 (9.09%)	
Fellow	3 (7.5%)	2 (5.13%)	0 (0.0%)	
Medical Student	9 (22.5%)	13 (33.33%)	0 (0.0%)	
Professor	2 (5.0%)	0 (0.0%)	0 (0.0%)	
Resident	24 (60.0%)	20 (51.28%)	10 (90.91%)	
	N = 40	N = 39	N = 11	
**In which context you met your mentor?**				0.12
academic context	17 (40.48%)	12 (27.91%)	1 (9.09%)	
dedicated pairing programs	0 (0.0%)	1 (2.33%)	1 (9.09%)	
surgical course	3 (7.14%)	1 (2.33%)	0 (0.0%)	
workplace	22 (52.38%)	29 (67.44%)	9 (81.82%)	

	N = 42	N = 43	N = 11	
**Did you receive surgical mentoring?**				0.541
Yes	36 (64.29%)	39 (72.22%)	10 (76.92%)	
No	20 (35.71%)	15 (27.78%)	3 (23.08%)	
	N = 56	N = 54	N = 13	
**Did you receive clinical mentoring?**				0.107
Yes	17 (30.36%)	21 (38.89%)	8 (61.54%)	
No	39 (69.64%)	33 (61.11%)	5 (38.46%)	
	N = 56	N = 54	N = 13	
**Were you mentored on research?**				0.355
Yes	19 (33.93%)	18 (33.33%)	7 (53.85%)	
No	37 (66.07%)	36 (66.67%)	6 (46.15%)	
	N = 56	N = 54	N = 13	
**Were you mentored on non-technical skills?**				**<0.001**
Yes	4 (7.14%)	19 (35.19%)	5 (38.46%)	
No	52 (92.86%)	35 (64.81%)	8 (61.54%)	
	N = 56	N = 54	N = 13	
**Which skills are essential to a mentee to deserve to be mentored and take advantage of the relationship?**				**0.043**
academic skills	4 (8.89%)	0 (0.0%)	2 (20.0%)	
non-technical skills	25 (55.56%)	35 (68.63%)	4 (40.0%)	
technical skills	16 (35.56%)	16 (31.37%)	4 (40.0%)	
	N = 45	N = 51	N = 10	
**Which is the best moment of the career to find a mentor?**				0.909
fellowship	2 (4.44%)	2 (3.92%)	1 (9.09%)	
it does not matter	4 (8.89%)	5 (9.8%)	1 (9.09%)	
medical school	6 (13.33%)	6 (11.76%)	0 (0.0%)	
residency	33 (73.33%)	38 (74.51%)	9 (81.82%)	
	N = 45	N = 51	N = 11	
**Do you believe that the mentor-mentee relationship should necessarily be based on a strong personal connection?**				0.9
Yes	28 (62.22%)	34 (66.67%)	7 (63.64%)	
No	17 (37.78%)	17 (33.33%)	4 (36.36%)	
	N = 45	N = 51	N = 11	
**Do you believe that a mentor should work in the same hospital of the mentee?**				0.756
Yes	32 (71.11%)	38 (74.51%)	7 (63.64%)	
No	13 (28.89%)	13 (25.49%)	4 (36.36%)	
	N = 45	N = 51	N = 11	
**Which area do you believe would benefit more from a shoulder-to-shoulder mentorship?**				0.185
academic/scientific skills	5 (10.87%)	1 (1.96%)	1 (9.09%)	
career progression	4 (8.7%)	7 (13.73%)	4 (36.36%)	
mentoring skill	1 (2.17%)	2 (3.92%)	0 (0.0%)	
follow evidence-based practices	1 (2.17%)	1 (1.96%)	0 (0.0%)	
life-work balance	0 (0.0%)	1 (1.96%)	0 (0.0%)	
networking	0 (0.0%)	0 (0.0%)	1 (9.09%)	
non-technical skills	5 (10.87%)	6 (11.76%)	0 (0.0%)	
surgical skills	30 (65.22%)	33 (64.71%)	5 (45.45%)	
	N = 46	N = 51	N = 11	

**At which moment shoulder to shoulder mentorship would work best?**				0.938
attending surgeon	1 (2.17%)	2 (3.92%)	0 (0.0%)	
fellowhip	4 (8.7%)	3 (5.88%)	0 (0.0%)	
medical student	4 (8.7%)	3 (5.88%)	0 (0.0%)	
residency	37 (80.43%)	43 (84.31%)	11 (100.0%)	
	N = 46	N = 51	N = 11	
**Do you believe that a remote mentorship could be effective?**				0.732
Yes	29 (63.04%)	27 (55.1%)	6 (60.0%)	
No	17 (36.96%)	22 (44.9%)	4 (40.0%)	
	N = 46	N = 49	N = 10	
**Which area you believe would benefit more from remote mentorship?**				0.584
academic/scientific skills	17 (37.78%)	24 (47.06%)	4 (36.36%)	
career progression	5 (11.11%)	8 (15.69%)	2 (18.18%)	
mentoring skills	1 (2.22%)	1 (1.96%)	0 (0.0%)	
follow evidence-based practices	3 (6.67%)	1 (1.96%)	0 (0.0%)	
networking	5 (11.11%)	10 (19.61%)	2 (18.18%)	
non-technical skills	8 (17.78%)	5 (9.8%)	1 (9.09%)	
surgical skills	3 (6.67%)	2 (3.92%)	2 (18.18%)	
work-life balance	3 (6.67%)	0 (0.0%)	0 (0.0%)	
	N = 45	N = 51	N = 11	
**In terms of clinical and surgical skills: how do you or did you take advantage of your mentor?**				0.104
clinical management of complications	6 (14.29%)	11 (22.0%)	0 (0.0%)	
be a mentors myself	3 (7.14%)	5 (10.0%)	4 (50.0%)	
intraoperative decision making	11 (26.19%)	17 (34.0%)	2 (25.0%)	
mental rehearsal	7 (16.67%)	7 (14.0%)	0 (0.0%)	
preoperative surgical decision making	15 (35.71%)	10 (20.0%)	2 (25.0%)	
	N = 42	N = 50	N = 8	
**How do your mentor opinions impact your daily practice?**				0.174
I carefully consider its opinion in my decision making process and then I make my call	36 (90.0%)	35 (74.47%)	7 (77.78%)	
I promptly change my practice if I get a different insight/opinion from my mentor	4 (10.0%)<br>N = 40	12 (25.53%)<br>N = 47	2 (22.22%)<br>N = 9	
**Do you believe that mental wellbeing of young trauma and emergency surgeons, especially related to tough clinical scenarios and life-work balance, can be positively influenced by having a mentor?**				0.766
Yes	39 (97.5%)	45 (93.75%)	11 (100.0%)	
No	1 (2.5%)	3 (6.25%)	0 (0.0%)	
	N = 40	N = 48	N = 11	
**Do you feel this area of study should be expanded in Europe?**	39 (97.5%)	44 (91.67%)	10 (90.91%)	0.378
Yes	1 (2.5%)	4 (8.33%)	1 (9.09%)	
No	N = 40	N = 48	N = 11	
**Do you believe that the mentor-mentee relationship can be oriented by institutional programs?**				>0.999
Yes	37 (92.5%)	45 (93.75%)	11 (100.0%)	
No	3 (7.5%)	3 (6.25%)	0 (0.0%)	
	N = 40	N = 48	N = 11	

**Should universities be in charge of pairing mentors and mentees during surgical career?**				0.947
No	7 (17.5%)	6 (12.5%)	1 (9.09%)	
Yes for medical students	3 (7.5%)	5 (10.42%)	0 (0.0%)	
Yes for surgery residents	23 (57.5%)	25 (52.08%)	7 (63.64%)	
Yes for surgical fellows	0 (0.0%)	2 (4.17%)	0 (0.0%)	
Yes for young attending	1 (2.5%)	1 (2.08%)	0 (0.0%)	
Yes regardless of the status	6 (15.0%)	9 (18.75%)	3 (27.27%)	
	N = 40	N = 48	N = 11	
**Should scientific societies be in charge of pairing mentors and mentees during surgical career?**				0.754
No	8 (20.51%)	12 (25.0%)	1 (9.09%)	
Yes for surgery residents	15 (38.46%)	13 (27.08%)	6 (54.55%)	
Yes for surgical fellows	5 (12.82%)	11 (22.92%)	2 (18.18%)	
Yes for young attending	3 (7.69%)	2 (4.17%)	0 (0.0%)	
Yes regardless of the status	8 (20.51%)	10 (20.83%)	2 (18.18%)	
	N = 39	N = 48	N = 11	

S3.4 Group comparison according to the area of European areas

Variable	Southern<br>N = 85	Western-Central<br>N = 24	Northern<br>N = 11	Eastern<br>N = 3	p-Value
**Age**	38.52 (± 10.14)<br>Range: (26.0 ; 65.0)<br>N = 85	47.42 (± 11.01)<br>Range: (30.0 ; 63.0)<br>N = 24	45.55 (± 12.93)<br>Range: (28.0 ; 68.0)<br>N = 11	47.67 (± 12.34)<br>Range: (34.0 ; 58.0)<br>N = 3	**0.002**
**Position**					**0.003**
Resident	29 (34.12%)	5 (20.83%)	3 (27.27%)	0 (0.0%)	
Surgeon in established practice	29 (34.12%)	17 (70.83%)	8 (72.73%)	2 (66.67%)	
Surgeon in transition to independent practice	27 (31.76%)	2 (8.33%)	0 (0.0%)	1 (33.33%)	
	N = 85	N = 24	N = 11	N = 3	
**Speciality**					**<0.001**
EGS	44 (51.76%)	2 (8.33%)	7 (63.64%)	1 (33.33%)	
Skeletal trauma	1 (1.18%)	11 (45.83%)	0 (0.0%)	1 (33.33%)	
Visceral Trauma	40 (47.06%)	11 (45.83%)	4 (36.36%)	1 (33.33%)	
	N = 85	N = 24	N = 11	N = 3	
**Type of hospital in which you currently work**					0.148
large referral academic hospital	59 (69.41%)	15 (62.5%)	11 (100.0%)	3 (100.0%)	
rural community	3 (3.53%)	3 (12.5%)	0 (0.0%)	0 (0.0%)	
urban community	23 (27.06%)	6 (25.0%)	0 (0.0%)	0 (0.0%)	
	N = 85	N = 24	N = 11	N = 3	
**Have you ever had a mentor?**					0.118
Yes	62 (72.94%)	16 (66.67%)	11 (100.0%)	3 (100.0%)	
No	23 (27.06%)	8 (33.33%)	0 (0.0%)	0 (0.0%)	
	N = 85	N = 24	N = 11	N = 3	
**Have you had one comprehensive mentor?**					0.595
Yes	40 (50.63%)	8 (34.78%)	5 (45.45%)	1 (33.33%)	
No	39 (49.37%)	15 (65.22%)	6 (54.55%)	2 (66.67%)	
	N = 79	N = 23	N = 11	N = 3	
**Have you had different mentors on different rotations during your**					0.204
**residency?**	55 (69.62%)	13 (56.52%)	10 (90.91%)	2 (66.67%)	
Yes	24 (30.38%)	10 (43.48%)	1 (9.09%)	1 (33.33%)	
No	N = 79	N = 23	N = 11	N = 3	
**At which stage of your career did you first meet your**					0.281
**mentor?**	6 (9.52%)	1 (6.25%)	0 (0.0%)	0 (0.0%)	
Attending Surgeon	5 (7.94%)	0 (0.0%)	0 (0.0%)	0 (0.0%)	
Fellow	20 (31.75%)	1 (6.25%)	1 (11.11%)	0 (0.0%)	
Medical Student	2 (3.17%)	0 (0.0%)	0 (0.0%)	0 (0.0%)	
Professor	30 (47.62%)	14 (87.5%)	8 (88.89%)	2 (100.0%)	
Resident	N = 63	N = 16	N = 9	N = 2	
**In which context you met your mentor?**					0.328
academic context	25 (37.88%)	3 (17.65%)	1 (10.0%)	1 (33.33%)	
dedicated pairing programs	1 (1.52%)	1 (5.88%)	0 (0.0%)	0 (0.0%)	
surgical course	4 (6.06%)	0 (0.0%)	0 (0.0%)	0 (0.0%)	
workplace	36 (54.55%)	13 (76.47%)	9 (90.0%)	2 (66.67%)	
	N = 66	N = 17	N = 10	N = 3	
**Did you receive surgical**					0.392
**mentoring?**	59 (69.41%)	14 (58.33%)	9 (81.82%)	3 (100.0%)	
Yes	26 (30.59%)	10 (41.67%)	2 (18.18%)	0 (0.0%)	
No	N = 85	N = 24	N = 11	N = 3	
**Did you receive clinical**					0.843
**mentoring?**	34 (40.0%)	7 (29.17%)	4 (36.36%)	1 (33.33%)	
Yes	51 (60.0%)	17 (70.83%)	7 (63.64%)	2 (66.67%)	
No	N = 85	N = 24	N = 11	N = 3	
**Were you mentored on**					0.636
**research?**	33 (38.82%)	8 (33.33%)	2 (18.18%)	1 (33.33%)	

Yes	52 (61.18%)	16 (66.67%)	9 (81.82%)	2 (66.67%)	
No	N = 85	N = 24	N = 11	N = 3	
**Were you mentored on non-technical skills?**	23 (27.06%)	3 (12.5%)	1 (9.09%)	1 (33.33%)	0.299
Yes	62 (72.94%)	21 (87.5%)	10 (90.91%)	2 (66.67%)	
No	N = 85	N = 24	N = 11	N = 3	
**Which skills are essential to a mentee to deserve to be mentored and take advantage of the relationship?**	3 (4.0%)	2 (11.11%)	1 (10.0%)	0 (0.0%)	0.54
academic skills	48 (64.0%)	8 (44.44%)	6 (60.0%)	2 (66.67%)	
non-technical skills	24 (32.0%)	8 (44.44%)	3 (30.0%)	1 (33.33%)	
technical skills	N = 75	N = 18	N = 10	N = 3	
**Which is the best moment of the career to find a mentor?**					0.752
fellowship	4 (5.33%)	0 (0.0%)	1 (10.0%)	0 (0.0%)	
it does not matter	8 (10.67%)	2 (10.53%)	0 (0.0%)	0 (0.0%)	
medical school	11 (14.67%)	1 (5.26%)	0 (0.0%)	0 (0.0%)	
residency	52 (69.33%)	16 (84.21%)	9 (90.0%)	3 (100.0%)	
	N = 75	N = 19	N = 10	N = 3	
**Do you believe that the mentor-mentee relationship should necessarily be based on a strong personal connection?**	50 (66.67%)	12 (63.16%)	5 (50.0%)	2 (66.67%)	0.805
Yes	25 (33.33%)	7 (36.84%)	5 (50.0%)	1 (33.33%)	
No	N = 75	N = 19	N = 10	N = 3	
**Do you believe that a mentor should work in the same hospital of the mentee?**	55 (73.33%)	13 (68.42%)	7 (70.0%)	2 (66.67%)	0.947
Yes	20 (26.67%)	6 (31.58%)	3 (30.0%)	1 (33.33%)	
No	N = 75	N = 19	N = 10	N = 3	
**Which area do you believe would benefit more from a shoulder-to-shoulder mentorship?**	3 (3.95%)	1 (5.26%)	3 (30.0%)	0 (0.0%)	0.577
academic/scientific skills	10 (13.16%)	4 (21.05%)	1 (10.0%)	0 (0.0%)	
career progression	3 (3.95%)	0 (0.0%)	0 (0.0%)	0 (0.0%)	
coaching skills	2 (2.63%)	0 (0.0%)	0 (0.0%)	0 (0.0%)	
follow evidence-based	1 (1.32%)	0 (0.0%)	0 (0.0%)	0 (0.0%)	
practices	0 (0.0%)	1 (5.26%)	0 (0.0%)	0 (0.0%)	
life-work balance	9 (11.84%)	1 (5.26%)	1 (10.0%)	0 (0.0%)	
networking	48 (63.16%)	12 (63.16%)	5 (50.0%)	3 (100.0%)	
non-technical skills	N = 76	N = 19	N = 10	N = 3	
surgical skills					
**At which moment shoulder to shoulder mentorship would work best?**					0.732
attending surgeon	3 (3.95%)	0 (0.0%)	0 (0.0%)	0 (0.0%)	
fellowhip	7 (9.21%)	0 (0.0%)	0 (0.0%)	0 (0.0%)	
medical student	6 (7.89%)	0 (0.0%)	1 (10.0%)	0 (0.0%)	
residency	60 (78.95%)	19 (100.0%)	9 (90.0%)	3 (100.0%)	
	N = 76	N = 19	N = 10	N = 3	
**Do you believe that a remote mentorship could be effective?**	46 (61.33%)	11 (61.11%)	3 (33.33%)	2 (66.67%)	0.426
Yes	29 (38.67%)	7 (38.89%)	6 (66.67%)	1 (33.33%)	
No	N = 75	N = 18	N = 9	N = 3	
**Which area you believe would benefit more from remote mentorship?**					0.309
academic/scientific skills	36 (48.0%)	4 (21.05%)	4 (40.0%)	1 (33.33%)	
career progression	7 (9.33%)	4 (21.05%)	4 (40.0%)	0 (0.0%)	
coaching skills	2 (2.67%)	0 (0.0%)	0 (0.0%)	0 (0.0%)	
follow evidence-based	3 (4.0%)	1 (5.26%)	0 (0.0%)	0 (0.0%)	
practices	13 (17.33%)	3 (15.79%)	1 (10.0%)	0 (0.0%)	
networking	8 (10.67%)	3 (15.79%)	1 (10.0%)	2 (66.67%)	
non-technical skills	4 (5.33%)	3 (15.79%)	0 (0.0%)	0 (0.0%)	

surgical skills	2 (2.67%)	1 (5.26%)	0 (0.0%)	0 (0.0%)	
work-life balance	N = 75	N = 19	N = 10	N = 3	
**In terms of clinical and surgical skills: how do you or did you take advantage of your mentor?**					0.25
management of complications	12 (16.9%)	2 (12.5%)	3 (30.0%)	0 (0.0%)	
clinical mentorship of youngers	5 (7.04%)	5 (31.25%)	1 (10.0%)	1 (33.33%)	
intraoperative decision making	22 (30.99%)	3 (18.75%)	3 (30.0%)	2 (66.67%)	
mental rehearsal	11 (15.49%)	3 (18.75%)	0 (0.0%)	0 (0.0%)	
preoperative surgical decision making	21 (29.58%)	3 (18.75%)	3 (30.0%)	0 (0.0%)	
	N = 71	N = 16	N = 10	N = 3	
**How do your mentor opinions impact your daily practice?**					0.904
I carefully consider its opinion in my decision making process and then I make my call	57 (81.43%)	12 (80.0%)	6 (75.0%)	3 (100.0%)	
I promptly change my practice if I get a different insight/opinion from my mentor	13 (18.57%)	3 (20.0%)	2 (25.0%)	0 (0.0%)	
	N = 70	N = 15	N = 8	N = 3	
**Do you believe that mental wellbeing of young trauma and emergency surgeons, especially related to tough clinical scenarios and life-work balance, can be positively influenced by having a mentor?**					>0.999
Yes	67 (94.37%)	17 (100.0%)	8 (100.0%)	3 (100.0%)	
No	4 (5.63%)	0 (0.0%)	0 (0.0%)	0 (0.0%)	
	N = 71	N = 17	N = 8	N = 3	
**Do you feel this area of study should be expanded in Europe?**					0.439
Yes	66 (92.96%)	17 (100.0%)	7 (87.5%)	3 (100.0%)	
No	5 (7.04%)	0 (0.0%)	1 (12.5%)	0 (0.0%)	
	N = 71	N = 17	N = 8	N = 3	
**Do you believe that the mentor-mentee relationship can be oriented by institutional programs?**					0.802
Yes	65 (91.55%)	17 (100.0%)	8 (100.0%)	3 (100.0%)	
No	6 (8.45%)	0 (0.0%)	0 (0.0%)	0 (0.0%)	
	N = 71	N = 17	N = 8	N = 3	
**Should universities be in charge of pairing mentors and mentees during surgical career?**					0.683
No	9 (12.68%)	2 (11.76%)	3 (37.5%)	0 (0.0%)	
Yes for medical students	6 (8.45%)	1 (5.88%)	1 (12.5%)	0 (0.0%)	
Yes for surgery residents	40 (56.34%)	8 (47.06%)	4 (50.0%)	3 (100.0%)	
Yes for surgical fellows	2 (2.82%)	0 (0.0%)	0 (0.0%)	0 (0.0%)	
Yes for young attending	2 (2.82%)	0 (0.0%)	0 (0.0%)	0 (0.0%)	
Yes regardless of the status	12 (16.9%)	6 (35.29%)	0 (0.0%)	0 (0.0%)	
	N = 71	N = 17	N = 8	N = 3	
**Should scientific societies be in charge of pairing mentors and mentees during surgical career?**					0.415
No	12 (17.14%)	4 (23.53%)	5 (62.5%)	0 (0.0%)	
Yes for surgery residents	23 (32.86%)	8 (47.06%)	1 (12.5%)	2 (66.67%)	
Yes for surgical fellows	14 (20.0%)	2 (11.76%)	1 (12.5%)	1 (33.33%)	
Yes for young attending	5 (7.14%)	0 (0.0%)	0 (0.0%)	0 (0.0%)	
Yes regardless of the status	16 (22.86%)	3 (17.65%)	1 (12.5%)	0 (0.0%)	
	N = 70	N = 17	N = 8	N = 3	
